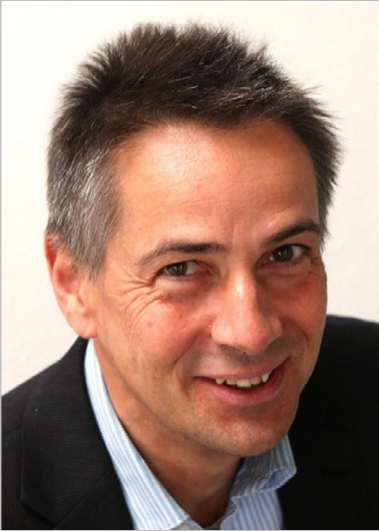# Synthetic Photoelectrochemistry

**DOI:** 10.1002/anie.201913767

**Published:** 2020-04-06

**Authors:** Joshua P. Barham, Burkhard König

**Affiliations:** ^1^ Universität Regensburg Fakultät für Chemie und Pharmazie 93040 Regensburg Germany

**Keywords:** electrochemistry, mediators, photoelectrochemistry, photoelectrodes, photoredox catalysis

## Abstract

Photoredox catalysis (PRC) and synthetic organic electrochemistry (SOE) are often considered competing technologies in organic synthesis. Their fusion has been largely overlooked. We review state‐of‐the‐art synthetic organic photoelectrochemistry, grouping examples into three categories: 1) electrochemically mediated photoredox catalysis (e‐PRC), 2) decoupled photoelectrochemistry (dPEC), and 3) interfacial photoelectrochemistry (iPEC). Such synergies prove beneficial not only for synthetic “greenness” and chemical selectivity, but also in the accumulation of energy for accessing super‐oxidizing or ‐reducing single electron transfer (SET) agents. Opportunities and challenges in this emerging and exciting field are discussed.

## Graphical abstract

**Photochemistry with potential**: The synergy of photochemistry and electrochemistry in organic synthesis is beneficial not only for synthetic sustainability and chemical selectivity, but also in the accumulation of energy for accessing super‐oxidizing or ‐reducing single electron transfer agents. Examples of synthetic organic photoelectrochemistry are dissected into three categories: electrochemically mediated photoredox catalysis, decoupled photoelectrochemistry, and interfacial photoelectrochemistry.

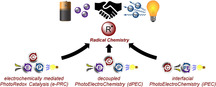

## 1 Introduction

Chemical synthesis by visible light is the fundamental process for biological photosynthesis on Earth. However, CO_2_ and H_2_O, and most organic molecules, do not absorb visible but ultraviolet light. Nature's solution is chlorophyll, a colored pigment that absorbs visible‐light energy to drive the process. Researchers have made efforts towards artificial photosynthesis with visible light ever since Giacomo Ciamician's vision in the turn of the 20th century (1912).^1^ By mimicking the concept of nature, but stripping down the complexity of interlinked photosystems into defined single‐molecule photocatalysts, researchers found that transition‐metal complexes such as bipyridyl complexes of Ru^II^ and Ir^III^ can harvest visible‐light photons to become powerful excited‐state single electron transfer (SET) agents for redox processes, and enjoy sufficiently long lifetimes (700–1100 ns)^2^ to undergo diffusion‐controlled redox events. Initial reports came as early as the 1980s,^3^ and the field of “visible light photoredox catalysis (PRC)” erupted in the turn of the 21st century.^2^, ^4^ In this context, seminal papers demonstrated the synthetic applications of Ru^II^ and Ir^III^ bipyridyl complexes.^5^, ^6^, ^7^

With sustainability and cost at the forefront of minds in academia and chemical industry,^8^ researchers were quick to challenge the presence of rare mid‐row transition metals with examples of organophotocatalysts such as eosin Y, Rose Bengal, and acridinium salts, as noted in seminal papers and reviews.4d, 4e, 4f, 4g, 9, 10, 11 Recently, the use of more sustainable transition‐metal‐based coordination compounds such as those of iron, nickel, and copper, whose excited‐state lifetimes are much shorter (rendering their application more challenging), are starting to receive attention.^12^ PRC is attractive for a variety of reasons reviewed elsewhere,^4^, ^13^ but arguably the biggest advantage is that use of visible light precludes direct excitation of substrates (leading to difficult‐to‐control high‐energy pathways and decomposition), selectively transferring energy to the photocatalyst chromophore.

Another vehicle for SET chemistry, which has been undergoing a renaissance in recent years, is synthetic organic electrochemistry (SOE). The application of electrical current in organic synthesis dates back as far as the Faraday and Kolbe electrolysis reactions from the 1830s to 1840s;^14^ far earlier than Ciamician's vision for artificial photosynthesis. A number of efforts^15^, ^16^, ^17^, ^18^, ^19^, ^20^ in the last two decades have brought SOE to the fore in organic chemistry.^21^ SOE is advantageous for several reasons that are well‐documented,^21^ but arguably the biggest advantage of SOE is the ability to dial in any potential, and the redox window is in theory only limited by the tolerance of the reaction solvent.

### 1.1 Visible‐Light Photoredox Catalysis: The Limits

A fundamental problem in visible‐light PRC is that the energy of processes is constrained by the energy of visible‐light photons (400–700 nm; ca. 1.8–3.1 eV). Inevitably, not all of this energy is accessible to the photocatalyst; losses occur due to intersystem crossing/non‐radiative pathways, which can account for up to approximately 0.6 eV in the case of Ru^II^ complexes.2a Ultimately, the energy available to a photocatalyst from excitation by a single visible‐light photon is typically insufficient for challenging chemical transformations such as the conversion of CO_2_ and H_2_O into glucose and water,^22^ or the direct SET activation of many moieties of interest to organic chemists. For example, SET oxidations of hydrocarbon C−H bonds,^23^ electron‐neutral/poor aromatic π‐systems,^24^ carbonyl groups,^25^ and ethers^25^ require potentials of +2.4–3.5 V vs. SCE, while reductions of aromatic π‐systems,^26^ aryl chlorides,^27^ and silyl halides^28^ require potentials of −2.6–3.4 V vs. SCE. In order to engage challenging moieties, visible‐light PRC has thus far relied on tricks that circumvent direct SET activation. For C−H or carbonyl activations, these can include 1) in situ generated radical or radical ions that undergo hydrogen atom transfer (HAT) chemistry,^29^ 2) excited states that directly engage in HAT chemistry,^30^ or 3) proton‐coupled electron transfer (PCET).^31^

Nature's solution to the “energy problem” is to accumulate the energies of multiple photons.^22^ Mimicry of such a technique has proven elusive to researchers until recent years. The concept of consecutive photoelectron transfer (conPET) was disclosed by König and co‐workers using a perylene diimide or RhB as the organophotocatalyst, to cleave C−X bonds that could not be cleaved by a single quantum of visible‐light energy.11d, 11e Following absorption of one quantum of visible‐light energy and then reduction by a sacrificial SET donor (e.g., Et_3_N), the formed radical anion absorbs the second quantum of visible‐light energy. Ultimately, a super‐electron donor is formed in situ by accumulation of visible photons. Although the subsequent chemistry may be redox‐neutral, the requirement for a sacrificial electron donor to ensure a sufficient concentration of photoexcitable radical anion is undesirable. This strategy may not be so general because it requires design of photocatalyst architectures that absorb visible light both in their ground state and in their radical ion state.

### 1.2 Synthetic Organic Electrochemistry: The Limits

A fundamental problem in SOE is that the conductivity of organic solvents is typically low (compared to aqueous systems). A high “ohmic drop” exists between the two separated electrodes, necessitating high cell potentials for useful reaction conversions. Such potentials may be high enough to encourage unselective, deleterious redox processes when applied to the organic substrate of interest. The cell potential is the sum of electrode potential and ohmic drop. By employing a high concentration of supporting electrolyte (such as *n*‐Bu_4_NPF_6_), the solution conductivity can be increased and the ohmic drop decreased;^32^ however, the amphiphilic electrolyte is generally (not always32b) difficult to separate from the desired product(s) after the reaction. A different strategy that allows reactions to proceed at milder electrode potentials is “mediated” electrolysis or “redox catalysis”.^21^, ^33^ Here, a mediator transports holes^17^ or electrons^27^, ^34^ to/from the electrode surface from/to the substrate. However, the redox power of mediators is limited to the redox potential of their radical ion or their ion forms.

## 2 Photoelectrochemical Organic Synthesis

**Figure 1:**
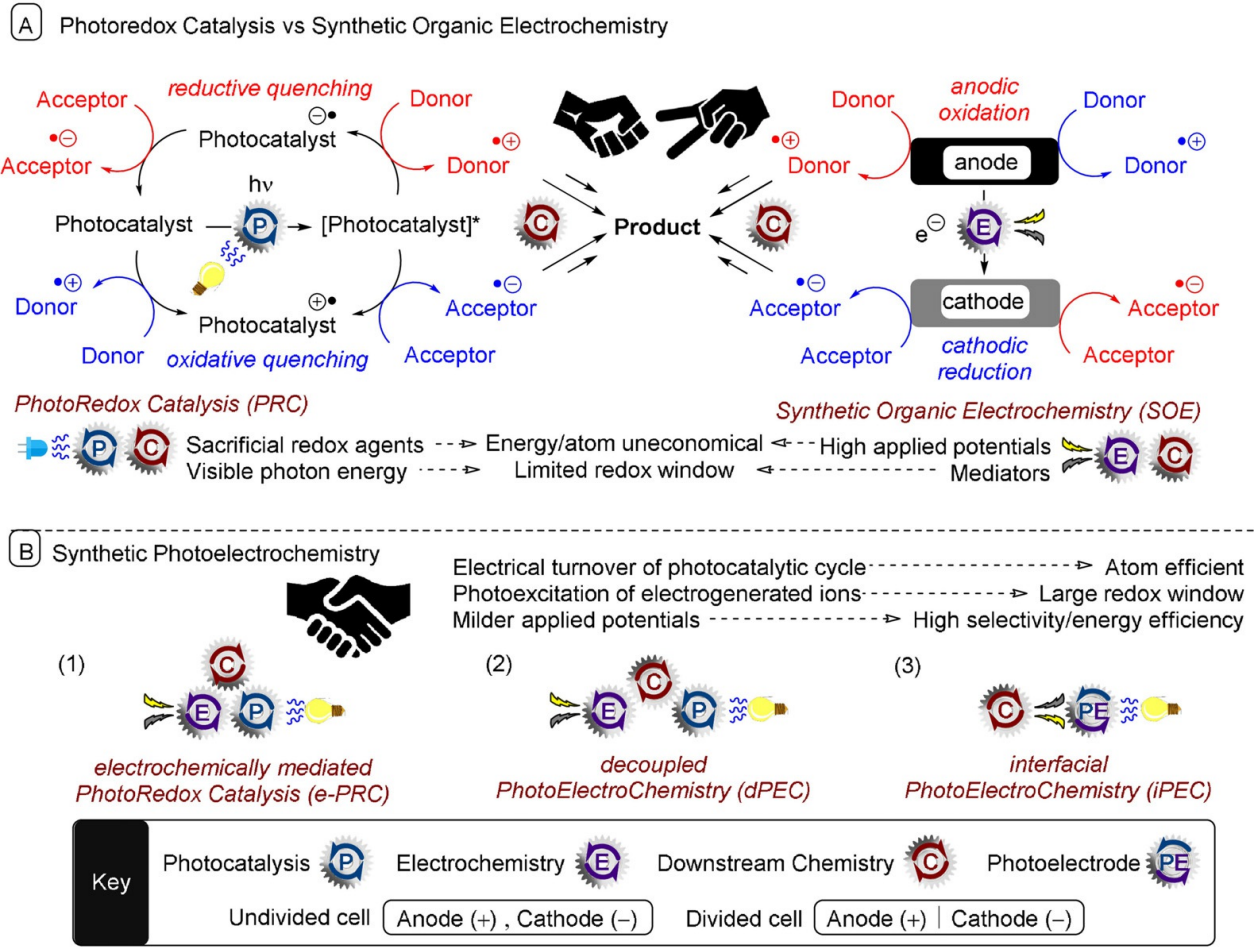
A) Comparison of photoredox catalysis (PRC) and synthetic organic electrochemistry (SOE). B) Types and benefits of synthetic photoelectrochemistry.

Visible‐light PRC and SOE have enjoyed a dramatic rise in popularity in the last decade, partly because of the drive towards green chemistry and sustainability but fundamentally because of their use as SET methods for straightforward access to organic free radicals that can be used in synthesis. In terms of their ability to perform redox chemistry, PRC and SOE are often thought of as competing technologies, and their fusion has thus far been largely overlooked (Figure 1). This Review explores synthetic photoelectrochemistry as the next evolutionary stage of PRC and SOE. State‐of‐the‐art examples are presented. For the purposes of this Review, we separate the examples into i) electrochemically mediated photoredox catalysis (e‐PRC), where the electrochemical and photochemical components have interdependent roles providing an explicit benefit within the chemical process; ii) decoupled photoelectrochemistry (dPEC), where electrochemical and photochemical components have separate, discrete roles; and iii) interfacial photoelectrochemistry (iPEC), where reactions occur at photoelectrode surfaces. In this Review, we focus only on the use of organic substrates and exclude the photoelectrolytic splitting of water and solar fuel production. For our justification of nomenclature and for recommendations to users of this technology, see Section 3.4.

### 2.1 Electrochemically Mediated Photoredox Catalysis (e‐PRC)

#### 2.1.1 Photoexcitation of Electrochemically Generated Ions

**Figure 2:**
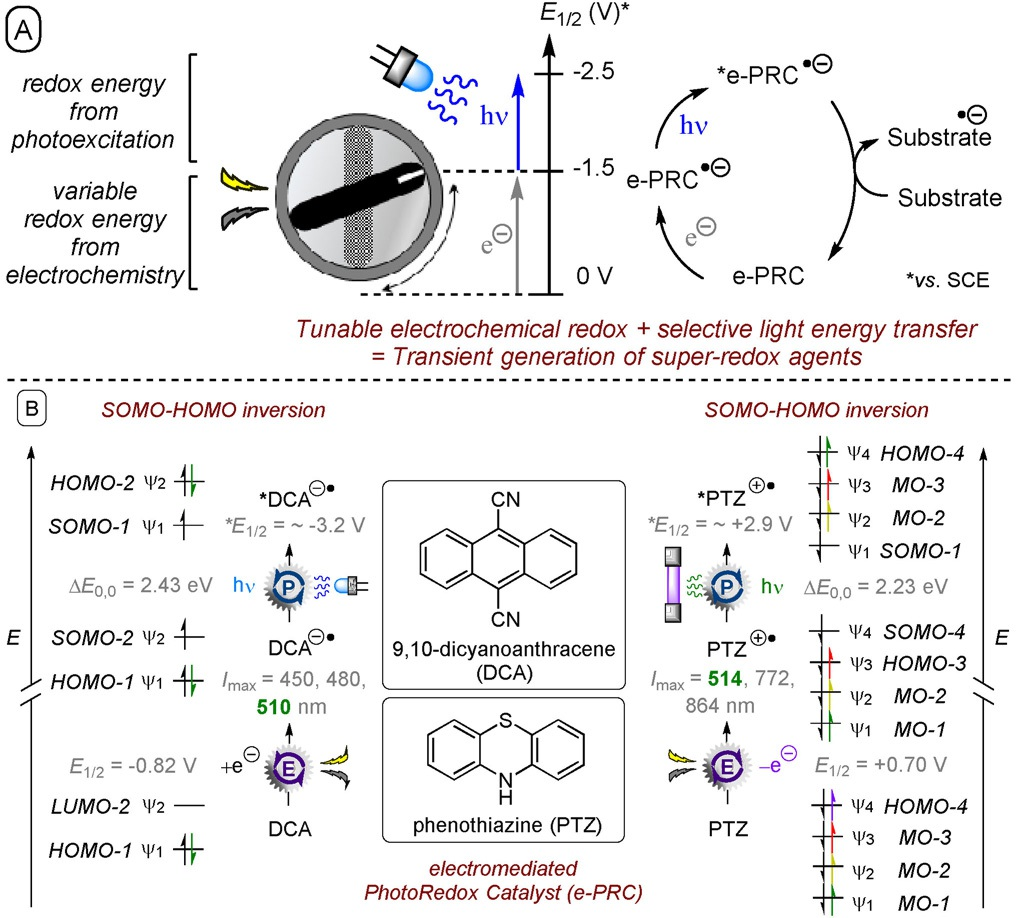
A) Conceptual redox energy level diagram for the photoexcitation of electrochemically generated ions in e‐PRC. B) SOMO–HOMO inversion concept for two electromediated photoredox catalysts (e‐PRCs).

One fundamental, exciting branch of e‐PRC is the photoexcitation of electrochemically generated ions.^35^, ^36^, ^37^, ^38^, ^39^ Here, a base redox energy level is provided by electrochemistry (e.g., a radical anion). Then, redox energy is provided from photoexcitation to generate super‐redox agents in a transient fashion (Figure 2). As the mediator is regenerated and accumulates both electrons and photons to overcome the activation energy barrier, the term “electromediated photoredox catalyst” (e‐PRC) can be coined. Considering the molecular orbital transitions of 9,10‐dicyanoanthracene (DCA) as an example of a recently reported^38^ reducing e‐PRC, the LUMO (ψ_2_) of DCA is first populated with an electron by cathodic current, thus becoming SOMO‐2 (ψ_2_) of DCA^.−^. Photoexcitation promotes an electron from the MO‐1 (ψ_1_) to the SOMO‐2 (ψ_2_), thus effecting SOMO–HOMO inversion.^38^ This also occurs in the complementary scenario with PTZ as an oxidizing e‐PRC;35a the removal of an electron by anodic current turns HOMO‐4 into SOMO‐4. An electron is then promoted from MO‐1 to SOMO‐4 by 514 nm light.^40^ In both cases, the e‐PRC becomes a doublet excited state.

**Figure 3:**
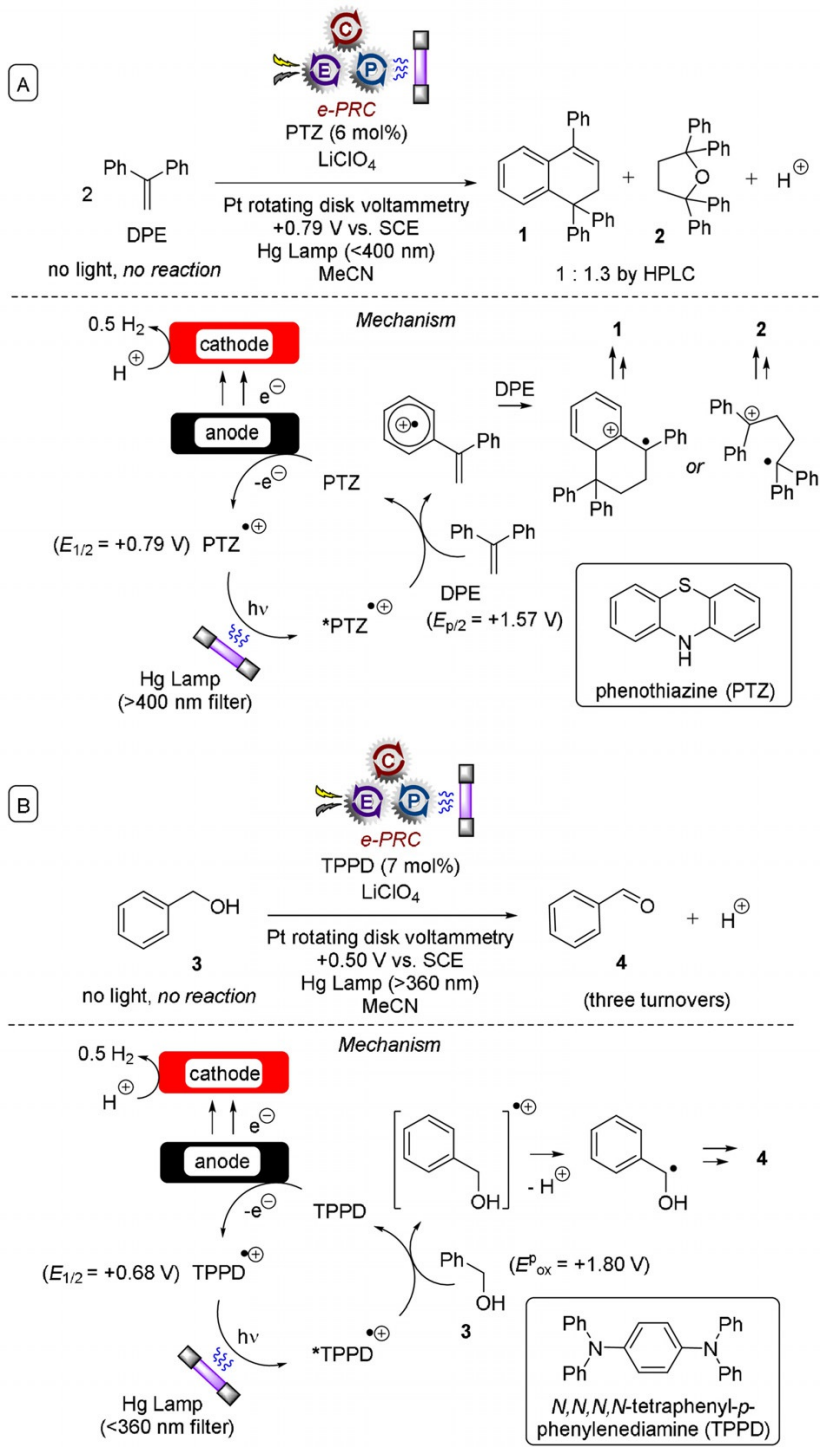
Early reports of photoelectrochemistry in organic synthesis: the photoexcitation of electrochemically generated ions.

The combination of photochemistry with electrochemistry within the context of organic synthesis was first disclosed by Moutet and Reverdy,^35^ who photoexcited electrochemically generated radical ions. Visible‐light photoexcitation (>400 nm) of the phenothiazine (PTZ) radical cation, generated electrochemically at controlled potential (*E*_1/2_ (PTZ)=+0.79 V vs. SCE),35c in the presence of 1,1‐diphenylethylene (DPE, *E* ^p/2^_ox_=+1.57 V vs. SCE),20j leads to oxidation of DPE and regeneration of phenothiazine (Figure 3 A).35a The DPE radical cation undergoes a [4+2] cycloaddition or 1,2‐addition with a second molecule of DPE, ultimately furnishing **1** or **2** upon further oxidation/reaction with H_2_O. No reaction of DPE with PTZ^.**+**^ occurred in the dark.

A few years later, Moutet and Reverdy reported that the electrogeneration of *N*,*N*,*N*′,*N*′‐tetraphenyl‐*p*‐phenylenediamine (TPPD) radical cations and their photoexcitation with UV light (366 nm) enabled oxidation of benzyl alcohol (**3**) to benzaldehyde (**4**; Figure 3 B).35b Interestingly, the oxidation of substituted benzyl alcohols, 1‐phenylethanol, or benzhydrol did not lead to the corresponding ketones, but rather to the symmetrical ethers. Here, e‐PRC is tentatively written because not enough details (yields, conversion) were reported to determine whether PTZ is catalytic in the first example. Whilst the second example could be considered to be the first report of e‐PRC, the process took place with only about three turnovers and the yield was not reported.

Following these^35^ and other early reports (generally investigated in an analytical/fundamental context),^36^, ^37^ photoelectrochemistry in organic synthesis did not receive attention until very recently. This naturally follows on from the resurgence of SET chemistry in organic synthesis thanks to PRC and SOE, which have been popularized in the last decade.

In terms of SET oxidation, among the most powerful photoredox catalysts are the acridinium salts (Mes‐Acr^+^) developed by Fukuzumi and co‐workers.^41^ Seminal papers by the group of Nicewicz employed these organophotocatalysts in the oxidation of alkenes to radical cations, which could be intercepted by nucleophiles in an anti‐Markovnikov‐type reaction.10a, 10b Moreover, direct oxidation of arenes was achieved, and their nucleophilic trapping with heterocyclic nucleophiles gave rise to a palladium‐free Buchwald–Hartwig‐type reaction.10d However, the former reaction was limited to styrenes or highly electron‐rich (trisubstituted) alkenes with a tethered nucleophile. The latter was limited to electron‐rich arenes (anisoles) because the redox potentials of mono‐/disubstituted alkenes (*E* ^p^_ox_=+2.37 V vs. SCE)^25^ and of benzene (+2.48 V vs. SCE)^39^ lie beyond the redox potential of the acridinium excited state (+2.06 V vs. SCE).4f

**Figure 4:**
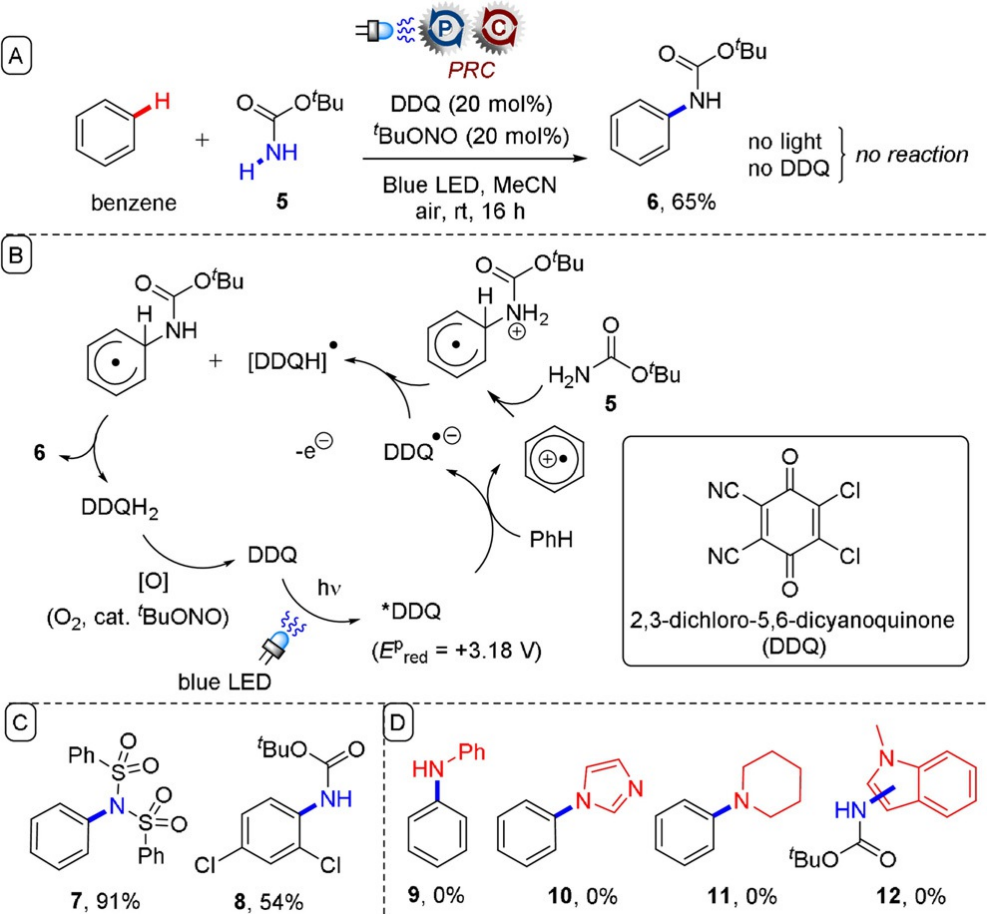
A) Oxidation of unactivated arenes under PRC using DDQ. B) Proposed mechanism. C) Examples from the substrate scope. D) Nucleophilic or arene partners that reacted with ground‐state DDQ.

One way that researchers overcame this limitation was by employing DDQ, which forms a very powerful excited triplet state (+3.18 V vs. SCE) that can engage unactivated or electron‐deficient arenes.^42^, ^43^ Photocatalytically generated arene radical cations can be intercepted by nucleophiles such as **5** to give aminated arenes such as **6** as demonstrated by the groups of König (Figure 4)^43^, ^44^ and others.^45^ However, DDQ is moderately expensive and is prone to promiscuous reactivity with arene substrates or amine nucleophiles (Figure 3 D), as well as other functional groups,^46^ through ground‐state oxidation chemistry.

**Figure 5:**
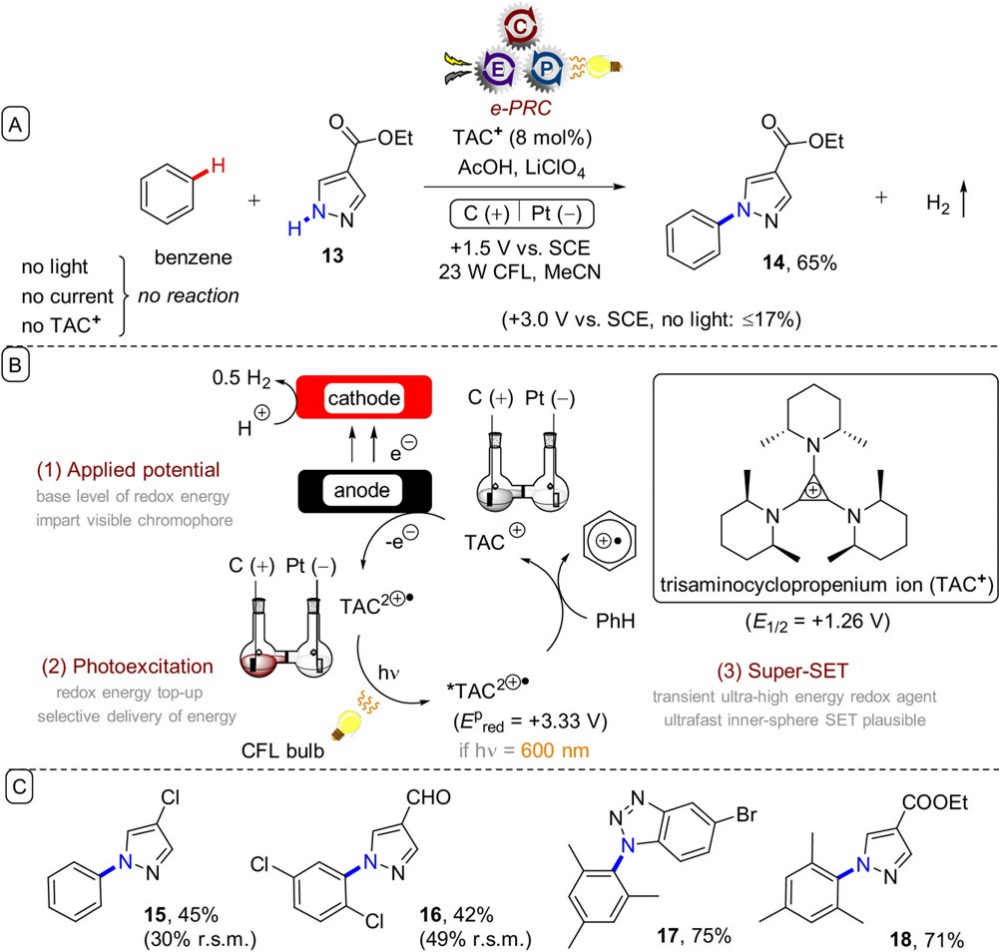
A) Direct oxidation of unactivated arenes by e‐PRC using photoexcited electrogenerated trisaminocyclopropenium dications. B) Proposed mechanism. C) Example scope.

Photoexcitation of electrochemically generated cations allows for redox potentials that are notably more positive than those achieved even with *Mes‐Acr^+^ and avoids the complications associated with PRC using DDQ. In an elegant and seminal e‐PRC example, Lambert and co‐workers reported the oxidation of unactivated arenes and their coupling with heterocyclic amines (Figure 5).^39^ Under anodic oxidation at a fixed potential (+1.50 V vs. SCE), colourless trisaminocyclopropenium cation (TAC^+^) was oxidized to its dication radical (TAC^.2+^; *E*_1/2_=1.26 V vs. SCE), which is strongly coloured. Excitation of TAC^.2+^ with visible light (ca. 600 nm) provided the superoxidant *TAC^.2+^ (*E*_1/2_=+3.33 V vs. SCE), which oxidized unactivated arenes to their radical cations. The remarkable potential of *TAC^.2+^ was rationalized by time‐dependent density functional theory (TD‐DFT) calculations, which revealed a SOMO–HOMO level inversion leaving a low‐lying hole in the HOMO. Ethyl 1*H*‐pyrazole‐4‐carboxylate (**13**) undergoes nucleophilic addition to the benzene radical cation, generating (upon loss of a proton) an aryl radical. Oxidation of the aryl radical, either by TAC^.2+^ or by the carbon (felt) anode, followed by loss of a proton, furnishes product **14**. Proton reduction was proposed as the corresponding cathodic half‐reaction, as gas bubbles were observed. Control reactions confirmed that no reaction occurred without light, current, or TAC. For comparison, direct electrolysis was performed at fixed potential (+3.0 V vs. SCE) and gave polymeric material, exemplifying the advantage of the mild conditions of e‐PRC. The reaction tolerated benzene and even chloroarenes to give products **15** and **16**, albeit in modest yield. Substituted triazoles, benzotriazoles, and purines were successful partners, affording products such as **17** and **18**. No oxidation of aldehyde‐, ketone‐, or ester‐bearing pyrazoles was observed. The expansion of scope to unactivated or electron‐deficient arenes represents a key advantage over Nicewicz's original report.10d

**Figure 6:**
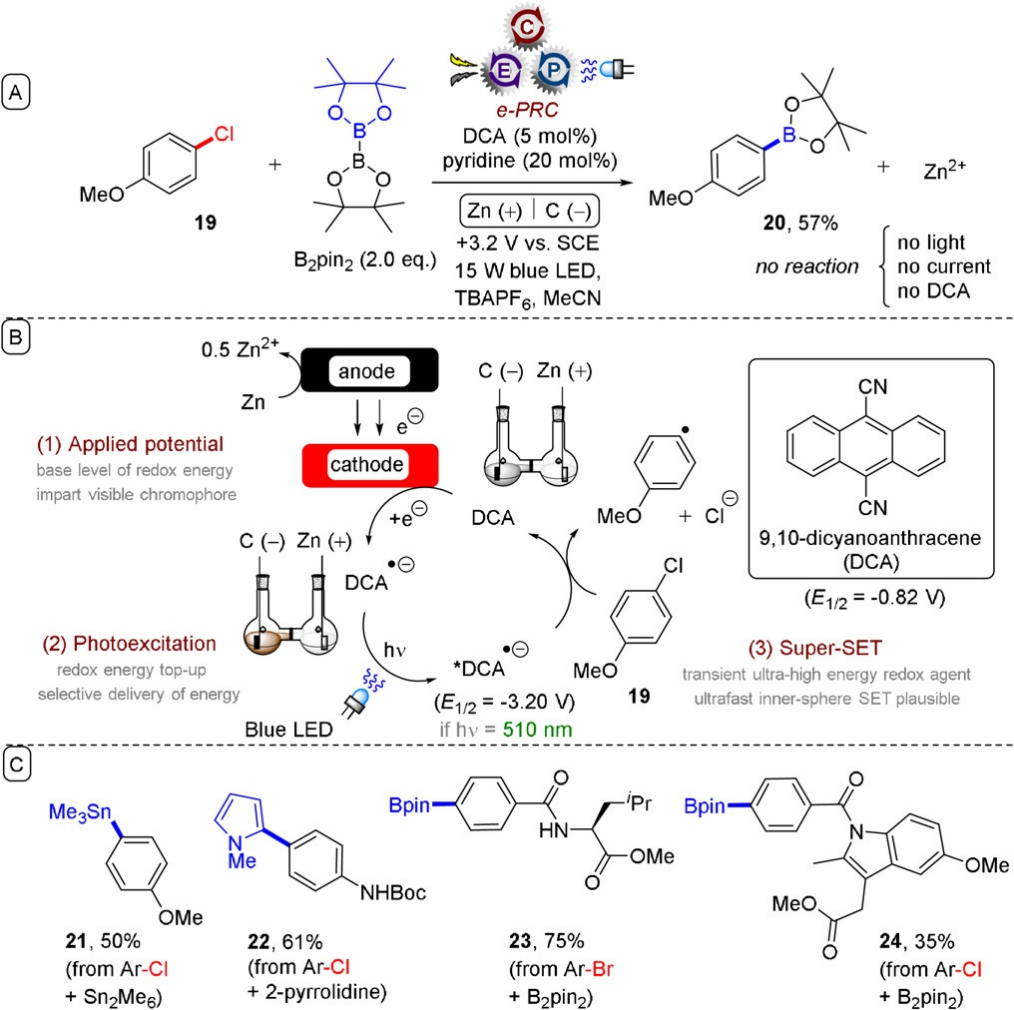
A) Direct reduction of electron‐rich chloroarenes by e‐PRC using photoexcited electrogenerated dicyanoanthracene radical anions. B) Proposed mechanism. C) Example scope.

In a complementary fashion, cathodic current can be used to generate radical anions photoexcited to generate superreductants. Lambert, Lin, and co‐workers reported the reduction of chloro‐ and bromoarenes such as **19** using photoexcited 9,10‐dicyanoanthracene radical anion (*DCA^.−^),^38^ itself generated by cathodic reduction of DCA by a porous carbon anode (Figure 6). The extraordinarily high reduction potential of −3.2 V vs. SCE was proposed to arise from a SOMO–HOMO level inversion and a highly unstable filled antibonding orbital, as confirmed by TD‐DFT calculations.^38^ The generated aryl halide radical anions fragment to afford halide anions and aryl radicals, the latter of which were successfully trapped with B_2_pin_2_, Sn_2_Me_6_, or heteroarenes to give products such as **21**–**24**. Oxidation of sacrificial Zn anode was proposed as the corresponding half‐reaction. The method provides a key advantage over palladium‐catalysed functionalizations used to generate similar products, which suffer when coupling partners contain Lewis basic groups (such as the precursor to **23**) as they alter the course of catalysis by coordination.

#### 2.1.2 Replacing Sacrificial Redox Agents with Current

**Figure 7:**
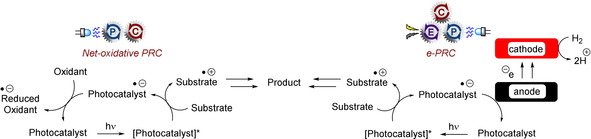
Comparison of net‐oxidative PRC and e‐PRC using anodic current for electrorecycling of the photocatalyst.

**Figure 8:**
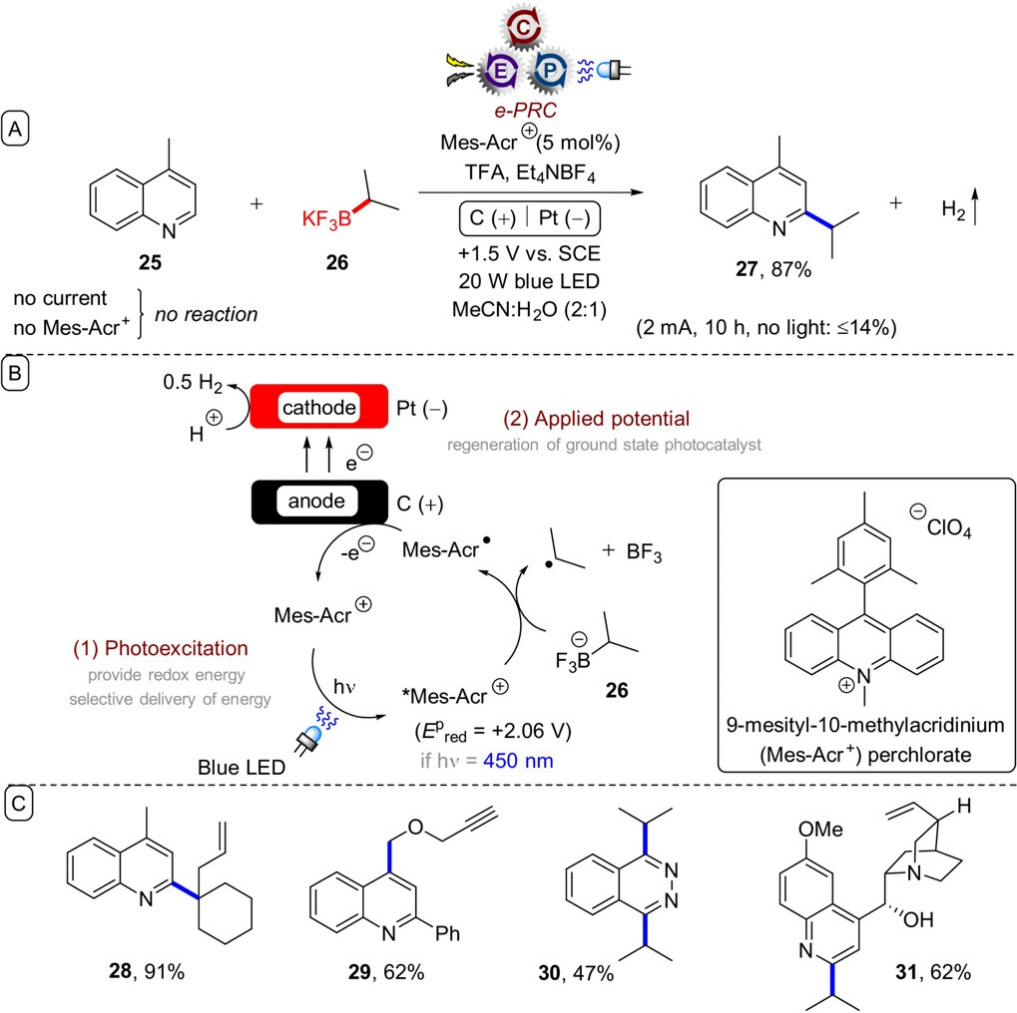
A) S_N_Ar reactions of unactivated aryl fluorides at ambient temperature and without base under e‐PRC. B) Proposed mechanism. C) Example scope.

Although the former subsection likely presented a more fundamental and potentially ground‐breaking advantage of e‐PRC in organic synthesis, replacement of sacrificial redox agents is another very important aspect offered by e‐PRC that appeals to a sustainability and industry perspective (Figure 7). Xu and co‐workers reported on the C−H alkylation of heteroarenes with trifluoroborates under e‐PRC (Figure 8).^47^ Photoexcited 9‐mesityl‐10‐methylacridinium (*Mes‐Acr^+^) is a potent oxidant (*E* ^p^_red_=+2.06 V vs. SCE) capable of SET oxidation of isopropyl trifluoroborate (**26**; *E* ^p^_ox_≈+1.50 V vs. SCE)^48^ to its secondary alkyl radical. The alkyl radical adds to the protonated quinoline **25**‐H^+^ in a Minisci‐type manner, which, followed by loss of a proton and SET oxidation (either by ground‐state Mes‐Acr^+^ (*E* ^p/2^_red_=−0.57 V vs. SCE) or by the anode) affords product **27**.

Mes‐Acr^+^ is regenerated by anodic oxidation of Mes‐Acr^.^ at a reticulated vitreous carbon (RVC) anode. A wide substrate scope of heteroarenes were employed, including isoquinolines, phenanthridines, phthalazines, benzothiazoles, acridines, and purines, affording products such as **28**–**31**. The reaction conditions tolerated secondary and tertiary amines as well as secondary alcohols and alkynes, which would all be prone to oxidation under direct electrolysis at high potentials.

**Figure 9:**
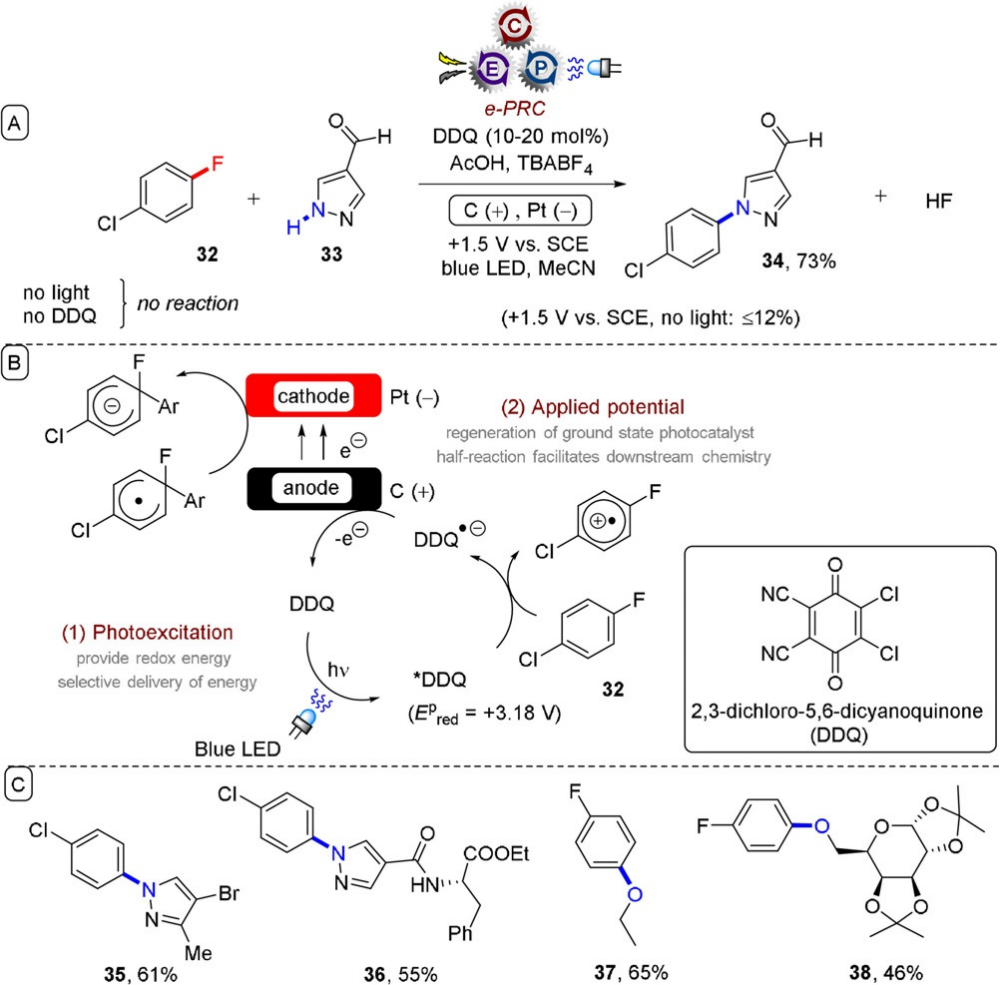
A) S_N_Ar reactions of unactivated aryl fluorides at ambient temperature and without base under e‐PRC. B) Proposed mechanism. C) Example scope.

Lambert and Huang reported S_N_Ar reactions of unactivated aryl fluorides under e‐PRC (Figure 9).^49^ Here, photoexcited 2,3‐dichloro‐5,6‐dicyanoquinone (DDQ) was sufficiently oxidizing (*E* ^p^_red_=+3.18 V vs. SCE) to engage chlorofluoroarenes such as **32** in SET oxidation. In terms of the heteroarene partner, the substrate scope was similar to that of the previous report involving the photoexcited dication *TAC^.2+^.^39^ Heteroarenes bearing aldehydes and esters were tolerated, affording products such as **35** and **36**. Alcohols such as ethanol and acetal‐protected galactose, as well as *tert*‐butyl carbamate, were also well‐tolerated as nucleophiles (products **37** and **38**). Redox potentials for the oxidation of polyhalogenated benzenes are unavailable in the literature, likely because they exceed the redox potential window of the solvent. It is interesting that although *TAC^.2+^ (*E* ^p^_red_= +3.33 V vs. SCE) is a more potent oxidant than *DDQ, it afforded a lower yield of **34**. This suggests that matching of redox potentials is not always a reliable predictor of successful SET chemistry and that other factors such as precomplexation of mediator and substrate (Section 3.3), might be important. Elsewhere, oxidation of unactivated alcohols was recently achieved under e‐PRC with riboflavin tetraacatate as the photocatalyst and thiourea as a HAT co‐catalyst.^50^ Here, the role of anodic current was to regenerate riboflavin from its dihydroquinone form.

### 2.2 Decoupled Photoelectrochemistry (dPEC)

**Figure 10:**
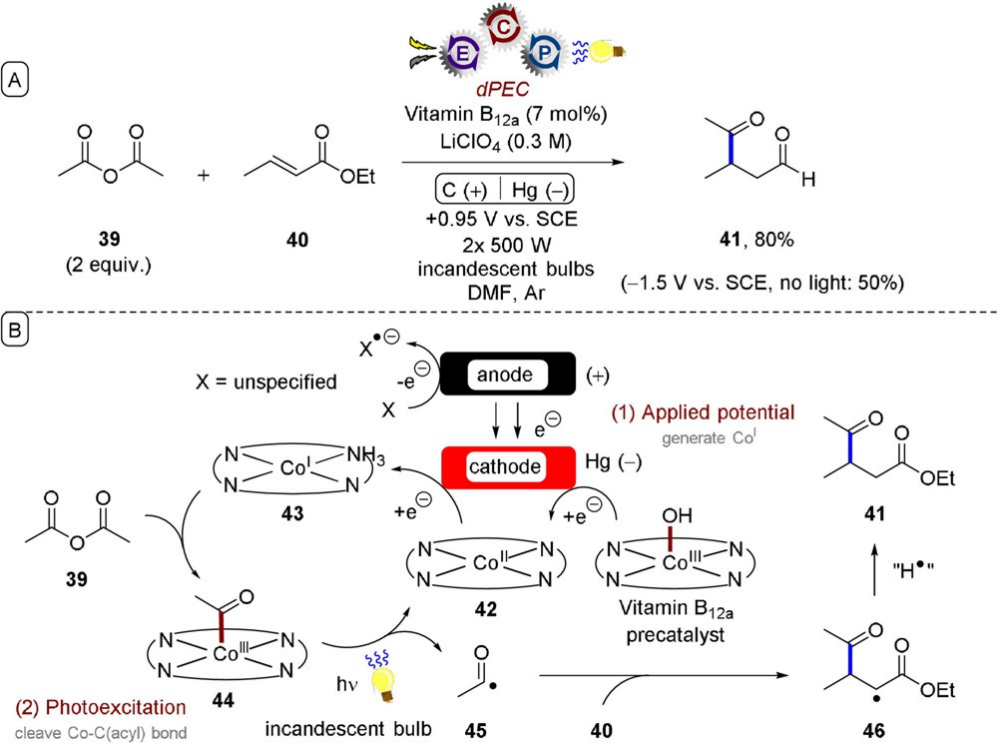
A) Photoelectrochemical 1,4‐addition of acyl groups under dPEC. B) Proposed mechanism.

Sheffold and Orlinski reported a photoelectrochemical 1,4‐addition of acyl groups to α,β‐unsaturated carbonyl compounds (Figure 10).^51^ Cathodic current reduced vitamin B_12a_ (Co^III^) or a Co^II^ macrocyclic complex **42** to give Co^I^ complex **43**, which reacted with anhydride **39**. Photochemical cleavage of the Co^III^−C bond of **44** presumably afforded an acyl radical **45**, primed for 1,4‐addition to **40** to give **46**. The authors claimed that HAT from the solvent to **46** yielded product **41**. SET reductions of **45** (to give an acyl anion primed for 1,4‐addition) or **46**, followed by proton transfer from the solvent, could not be ruled out. The authors did not specify the anodic half‐reaction or the anode materials. Here, photochemistry and electrochemistry were handled as discrete processes, representing the first example of decoupled photoelectrochemistry (dPEC).

**Figure 11:**
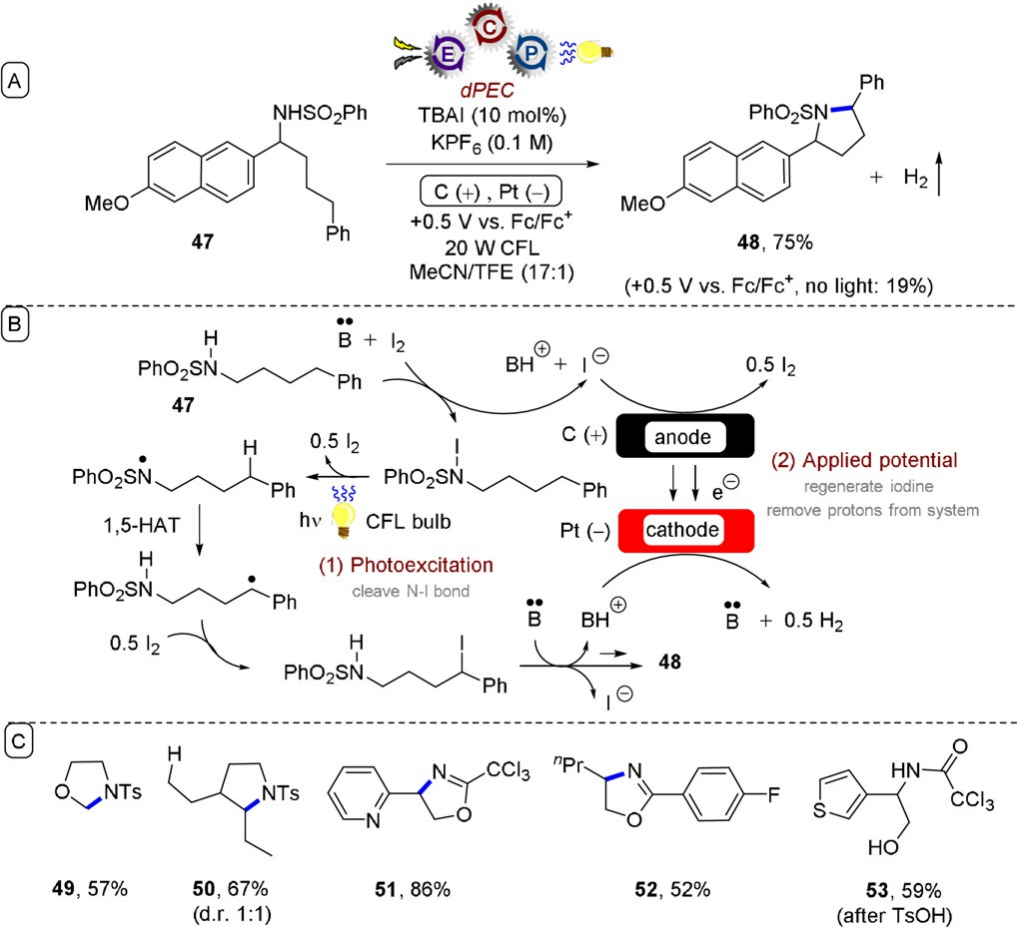
A) Hofmann–Löffler–Freytag amination of C(sp^3^)−H bonds under dPEC. B) Proposed mechanism. C) Example scope.

Stahl and Wang reported a Hofmann–Löffler–Freytag‐type (HLF) amination of C(sp^3^)−H bonds under dPEC.^52^ (Figure 11). Near‐UV photochemistry cleaved the N−I bond while the anodic potential oxidized iodide to molecular iodine (the cathodic reaction involved reduction of protons to hydrogen). The reaction successfully engaged both activated (benzylic, or adjacent to a heteroatom) and unactivated C(sp^3^)−H bonds (products **49** and **50**).

In addition to the HLF reactions of *N*‐alkyl sulfonamides to afford pyrrolidines, 2,2,2‐trichloroacetimidates (and benzimidates) were employed to afford oxazolines (products **51** and **52**). Various heterocycle‐bearing substrates were tolerated despite the anodic potential and in situ generated molecular iodine. Acid hydrolysis of the oxazolines gave rise to pharmaceutically valuable (protected) 1,2‐amino alcohols (product **53**). This work follows on from electrochemical HLF reactions reported by Muñiz,^53^ yet exhibits a key advantage in its use of low anodic potentials for the oxidation of iodide to molecular iodine. Such potentials are less positive than the redox potentials of electron‐rich arenes and other functional groups, and thus the mild conditions allow for excellent redox chemoselectivity. Stahl demonstrated^52^ that previously reported electron transfer/proton transfer/electron transfer (ET‐PT‐ET),^53^ proton‐coupled electron transfer (PCET),^54^ and bromide‐mediated electrochemical HLF reactions^55^ all failed to convert **47** into product **48**, instead yielding a complex mixture of products.

### 2.3 Interfacial Photoelectrochemistry (iPEC)

**Figure 12:**
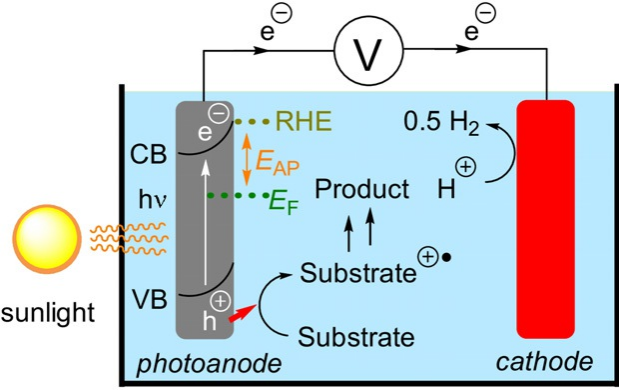
Schematic of a photoanode used for oxidation of organic compounds. RHE: relative Hydrogen electrode; *E*_AP_: applied potential; *E*_F_: Fermi level; CB: conduction band; VB: valence band.

**Figure 13:**
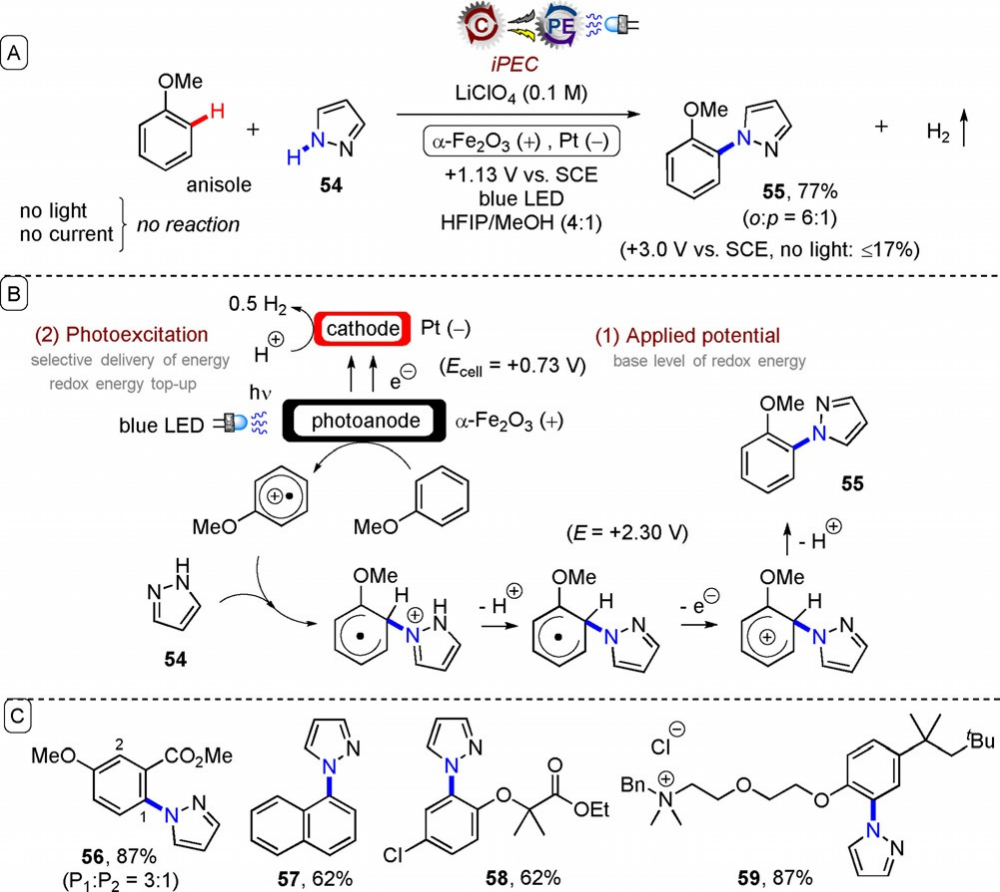
A) iPEC C−H amination of electron‐rich arenes with a hematite photoanode. B) Proposed mechanism. C) Example scope.

In interfacial photoelectrochemistry (iPEC), also known as “photoelectrocatalysis”, a photoelectrode is coated in a photoresponsive (typically, semiconductor) material whose band gap corresponds to the energy of a visible‐light photon. An applied or “bias” potential (*E*_AP_) is used to improve charge carrier separation upon irradiation (preventing recombination and generation of heat). For photoanodes, applied potential followed by irradiation promotes an electron from the valence band to the conductive band, generating a hole that is used for oxidation chemistry (Figure 12).^56^ Hu, Grätzel, and co‐workers recently reported the use of a photoelectrochemical cell in organic synthesis as an example of iPEC.^57^ After setting the photoelectrochemical cell at a fixed applied potential (+1.13 V vs. SCE), a hematite (α‐Fe_2_O_3_) photoanode was irradiated with blue LEDs and was rendered highly oxidizing (valence band=+2.30 V vs. SCE). Anisole was oxidized to its radical cation, primed to nucleophilic attack by a range of aromatic heterocycles (such as **54**) in an overall C−H amination of electron‐rich arenes to furnish products such as **55**–**59** (Figure 13). In the absence of light, higher applied potentials (+1.93 V vs. SCE) were required to access the desired chemistry and in decreased yield. Direct electrolysis (in the dark) with a conductive glassy carbon electrode (+1.73 V vs. SCE) gave poorer yields and side products that were absent when the photoelectrochemical cell and light were used.

The fact that the arene scope was limited to electron‐rich arenes, mirroring the original amination report of Nicewicz and co‐workers,10d is unsurprising considering that the hematite band gap (2.3 V vs. SCE) is similar to the redox potential of *Mes‐Acr^+^ (*E* ^p^_red_=+2.06 V vs. SCE). The markedly different *ortho*/*para* (*o*/*p*) selectivity between the two reports10d, 57 was attributed by the authors to the hexafluoroisopropanol solvent. The authors proposed that HFIP creates a hydrogen‐bonding network that favours substitution at the *ortho* position, and that the fundamental photoelectrochemical process proceeds through the same intermediates as the photochemical example.10d One possibility not yet considered is that precomplexation of acridinium photocatalyst and anisole (Section 3.3), or precomplexation of the anisole with the photoanode, may encourage stereoelectronic effects that bias the selectivity.

**Figure 14:**
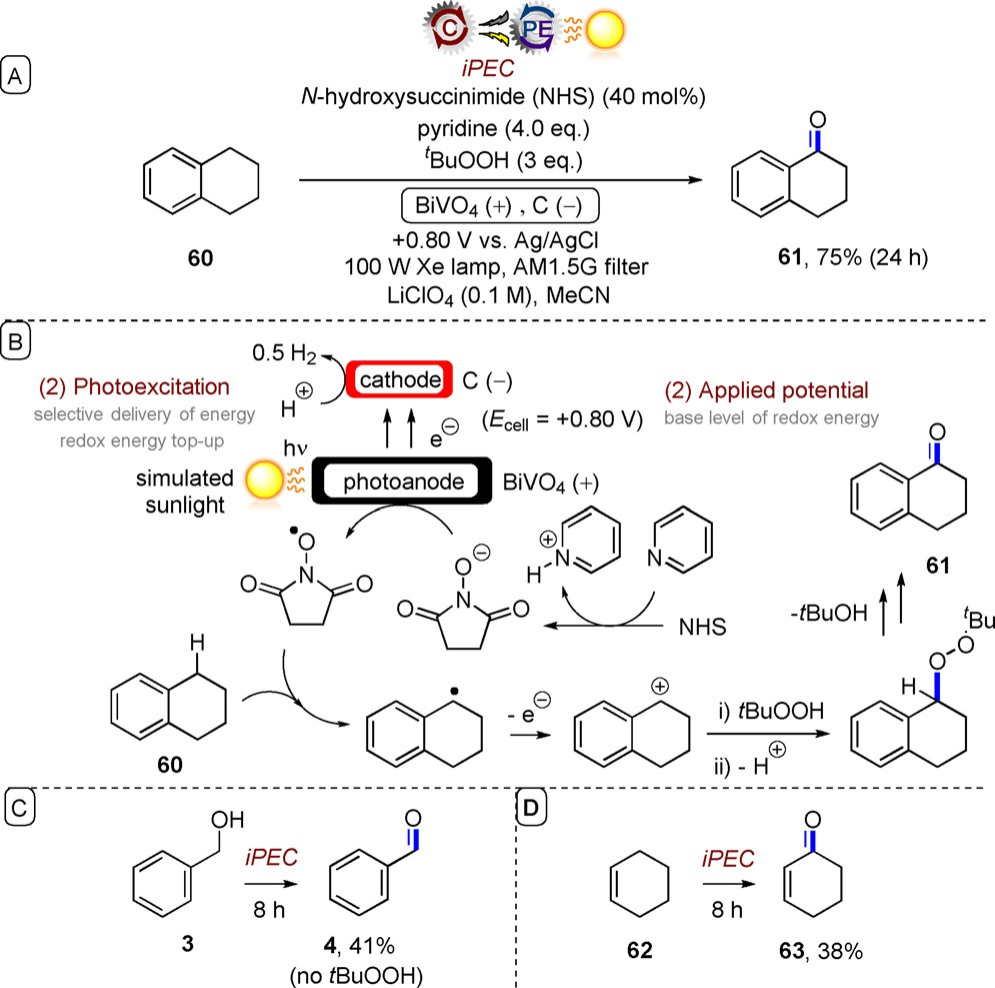
A) iPEC oxidation of simple organic compounds with a BiVO_4_ photoanode. B) Proposed mechanism. C) Benzyl alcohol iPEC oxidation. D) Cyclohexene iPEC oxidation.

Several reports of oxidation of simple organic molecules by iPEC exist, for example, alcohol oxidations.^58^ However, such reactions generally occur in aqueous solvent systems, and certain photoanode materials are known to undergo photocorrosion in aqueous solvent systems.^59^ Sammis, Berlinguette and co‐workers reported oxidations of tetralin (**60**), benzyl alcohol (**3**), and cyclohexene (**62**) in MeCN under iPEC using a BiVO_4_ photoanode and a 100 W Xe lamp fitted with an AM1.5G filter as simulated sunlight, to give products **61**, **4**, and **63**, respectively (Figure 14).^60^ *N*‐Hydroxysuccinimide (NHS) was employed as a soluble, transparent hole‐transfer mediator^15^, ^61^ between the photoanode and the substrates. For oxidations of **60** and **62**, it was necessary to employ *t*‐BuOOH as the external oxygen source. The same oxidations could be achieved under electrochemical potential only (*E*_cell_=+1.8 V vs. Ag/AgCl) with a glassy carbon anode/cathode, leading the authors to assume that this potential matched the pseudo‐standard potential of NHS. However, the authors noted that their iPEC method, which operates at a 1.0 V lower applied potential than the electrochemical cell, expects energy savings of 60 %. Although product yields were modest, the authors noted that the ability to perform organic synthesis at a solar‐to‐electricity efficiency (*η*=1.3 %) close to that of traditional photoelectrochemical water oxidation (*η*=1.7 %) is important because of the higher value of the organic products.

**Figure 15:**
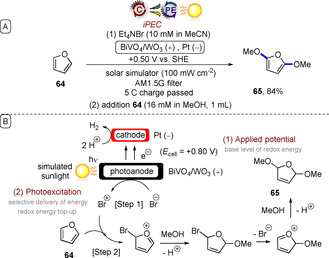
A) iPEC oxidative dimethoxylation of furan mediated by bromide ions using a BiVO_4_/WO_3_ photoanode. B) Proposed mechanism.

Related to this report is the iPEC C−H oxidation of cyclohexane by a WO_3_ photoanode^62^ and the iPEC oxidation of benzylic alcohols by a BiVO_4_/WO_3_ photoanode^63^ reported by Sayama and co‐workers, which both showed a drastic decrease in the applied potentials required for oxidation in the presence of light. Sayama further employed the BiVO_4_/WO_3_ photoanode in the iPEC oxidative dimethoxylation of furan **64** mediated by bromide ions (Figure 15).^64^ In the first step, oxidation of bromide anions by the photoanode afforded a pool of bromine cations. After 5 C of charge had passed and furan in MeOH had been added, the dimethoxylated product **65** was obtained in very good yield.

All of these reports exemplify the advantage of iPEC in leveraging the energy of visible light to offset the high applied electrode potentials otherwise needed, thus affording better selectivity and energy efficiency in chemical redox processes.^57^, ^60^, ^62^, ^63^, ^64^, ^65^ The initial modification of the photoelectrode with applied potential, followed by energy top‐up via the selective delivery of light energy, is conceptually similar to e‐PRC involving photoexcitations of electrochemically generated ions. The advantage of iPEC is that it does not rely on the generation of a chromophore in solution, and can directly engage substrates that do not absorb visible light. The disadvantage is that iPEC cannot reap the energy benefits of e‐PRC, which can access very high redox potentials (Figure 13 vs. Figure 5). While most examples of iPEC to date have dealt with simple chemical transformations, iPEC will undoubtedly occupy an important role in redox transformations of more complex organic substrates in the future.

## 3 Future Opportunities and Challenges

### 3.1 Practical Execution and Experimental Rigor

**Figure 16:**
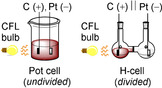
Typical custom‐built batch photoelectrochemical cells.

Thus far, the synthetic photoelectrochemistry examples reported herein have been conducted in custom‐built (transparent) electrochemical reaction vessels. These generally fall into two categories (Figure 16): a) an undivided glass “pot cell”/“beaker cell”/undivided glass voltammetry setup,^35^, ^47^, ^53^, ^58^, ^61^, ^63^ or b) a divided glass “H‐type” cell with a glass or membrane frit.^39^, ^49^, ^52^, ^63^ These are all standard academic reactors used for SOE,^66^ which can be easily irradiated with visible light. It is widely accepted that one of the drivers behind the renaissances of PRC and SOE in the last decade is the availability of reactor equipment. Indeed, visible‐light photoredox and synthetic organic electrochemical batch reactors have now been standardized and some are commercially available,^67^, ^68^, ^69^ addressing the long‐standing plague of practical irreproducibility in both fields. The design of suitable and standardized synthetic photoelectrochemical equipment will carry its own set of challenges, but fortunately, photoelectrochemical cells that have been developed for hydrogen production, such as the Cappicino PEC cell (EPFL Switzerland),^70^ the PortoCell (UPorto),^71^ and designs by Redoxme AB^72^ could be readily adapted for synthetic applications in organic solvents. Another challenge is the need for more rigorous control experiments (in absence of either light, applied potential, or e‐PRCs) to ensure that both the photochemical and electrochemical components are necessary and beneficial to the reaction.

### 3.2 Flow Photoelectrochemistry

Both PRC and SOE suffer upon scaling up in batch mode due to the physical constraints governing transfer of photons to the reaction or electrons to/from the reaction. The relationship between absorbance *A*, extinction coefficient *ϵ*, path length *l*, and molar concentration *c* is given by the Beer–Lambert law [Eqn. (1)]. Rearrangement to Equation (2) shows the exponential relationship between the transmitted intensity *I* and the absorbance *A*, which highlights the fundamental challenge faced in the scale‐up of photochemical processes. General theory predicts that for a typical 50.0 mm reaction with a photocatalyst loading of 1 mol % (0.5 mm) and *ϵ*=11280 m ^−1^ cm^−1^ (452 nm absorption band of Ru(bpy)_3_Cl_2_), 90 % of the light is absorbed at *l*=0.2 cm from the reactor surface.^73^ This tiny path length highlights the importance of the surface area to volume (SAVR) ratio in photochemical processes.

(1) $$A=\log_{10}\frac{I_{0}}{I}=\epsilon lc$$

(2) $$I=I_{0}e^{-\epsilon cl}=\;I_{0}e^{-\epsilon cl}$$

While SOE in macrobatch reactors has been achieved on an industrial scale, phenomena such as interelectrode ohmic drop, mass transfer, reaction selectivity, or environmental factors have presented barriers to various synthetic processes.^74^ In SOE, the rate‐limiting step of electrochemical reactions is generally how quickly the reagents can reach the proximity of the electrode surface (within which electron transfer can occur) by mass transfer, rather than the kinetics of the chemical reaction.^66^ The cell current *I*_cell_ (and, in turn, the cell's productivity) is given by a derivative of the Butler–Volmer equation [Eqn. (3)] and is related to the number of moles of reagent to be converted *n*, Faraday's constant *F*, electrode surface area *A* (cm^2^), mass transfer coefficient *k*_m_, and reagent concentration *c*. Hence, the cell productivity can be increased by increasing the electrode surface area and by mixing (increasing *k*_m_ by decreasing the size of the diffusion layer). The time‐dependent fractional conversion *X* of a mass‐transfer‐limited reaction of volume *V* is given by Equation (4).^66^ This demonstrates the key importance of efficient mixing (increasing *k*_m_) and largest possible electrode SAVR.

(3) $$I_{cell}=nFAk_{m}c$$

(4) $$X=1-exp\frac{-k_{m}At}{V}$$

Continuous flow (CF) is a globally recognized technology within chemical industries and academia^75^ that is especially useful in photochemistry^76^ and electrochemistry,^77^ because the flow of a reaction mixture through small‐diameter (μm–mm) channels 1) allows shorter path lengths for light transmission, 2) minimizes separation of electrodes (“ohmic drop”), allowing wasteful electrolytes to be eliminated or used in smaller amounts, 3) enhances mixing or user control over mixing by laminar or turbulent flow regimes, and 4) increases SAVR. Indeed, CF has even enabled multigram‐ to kilogram‐scale photochemical^78^ and SOE operations.^79^ Just as CF has enabled PRC and SOE separately, it is expected to be an enabling platform for synthetic photoelectrochemistry.

**Figure 17:**
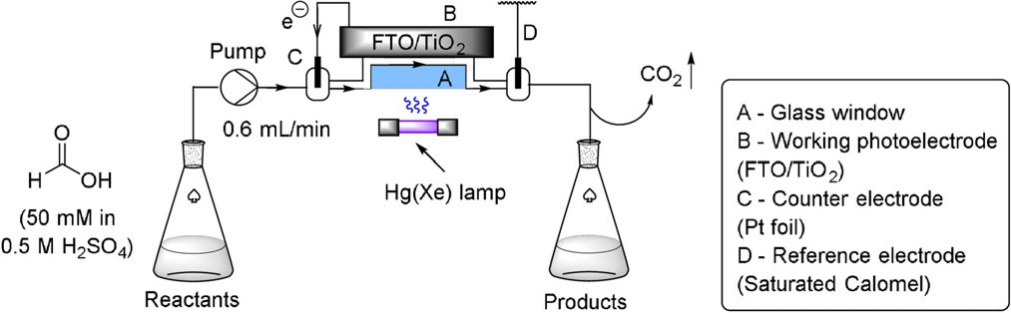
Continuous‐flow photoelectrochemical formic acid oxidation to CO_2_.

**Figure 18:**
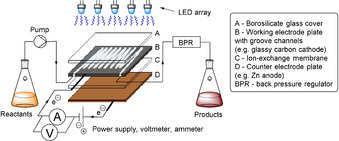
Conceptual photoelectrochemical flow reactor.

Reports of flow photoelectrochemistry have thus far focussed on simple chemical transformations. For example, Behm and co‐workers reported the oxidation of formic acid to CO_2_ by a photoanode in CF.^80^ A thin film of reaction mixture was pumped over a fluorine‐doped tin oxide(FTO)/TiO_2_ photoanode under irradiation from a Hg(Xe) (200 W) lamp (Figure 17). Such a configuration is suitable for certain chemical transformations, but may not be suitable for organic synthesis in general. This is due to shielding of the photoelectrode via absorption of UV light by reactants flowing atop it, facilitating potential side reactions/slow kinetics derived therefrom. Visible‐light e‐PRC or iPEC in CF has the advantage of selective delivery of light to the coloured e‐PRC mediator in the flow path or through the flow path to the photoelectrode, respectively. A conceptual CF photoelectrochemical reactor for synthesis is shown in Figure 18. Here, groove channels are etched into the working electrode, which is covered with a borosilicate glass window. An ion‐exchange membrane is sandwiched between the working electrode and counter electrode (here, a sacrificial counter electrode is assumed). The working electrode could be replaced with a photoelectrode for iPEC. Additional groove channels could be incorporated into/above the counter electrode if a solution‐phase half‐reaction is necessary.^81^

### 3.3 Precomplexation and Redox Processes beyond the Electrochemical Solvent Window

**Figure 19:**
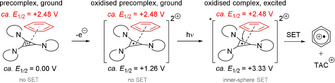
Herein proposed precomplexation of radical dication TAC^⋅2+^/benzene, photoexcitation, and quenching by ultrafast inner‐sphere SET.

Electrogenerated and photoexcited e‐PRCs (*PTZ^.**+**^, *DCA^.−^, *TAC^.2+^) discussed herein (Section 2.1.1) are rare, doublet excited states. The ultrashort lifetime of doublet excited states (fs to ps)^40^, ^82^ is shorter than the timeframe for diffusion control and should prohibit outer‐sphere SET events. While the mechanisms of such excited‐state processes are still unclear, precomplexation is likely responsible for ultrafast quenching (inner‐sphere SET) of e‐PRCs and successful reactions. For example, π–π stacking to generate a precomplex that is photoexcited has been proposed to explain reactions involving excited perylene diimide radical anions and arenes.11d, 83 Such a phenomenon may likewise rationalize Lambert's e‐PRC oxidation of unactivated arenes^39^ by *TAC^.2+^ as an e‐PRC (Figure 19).

**Figure 20:**
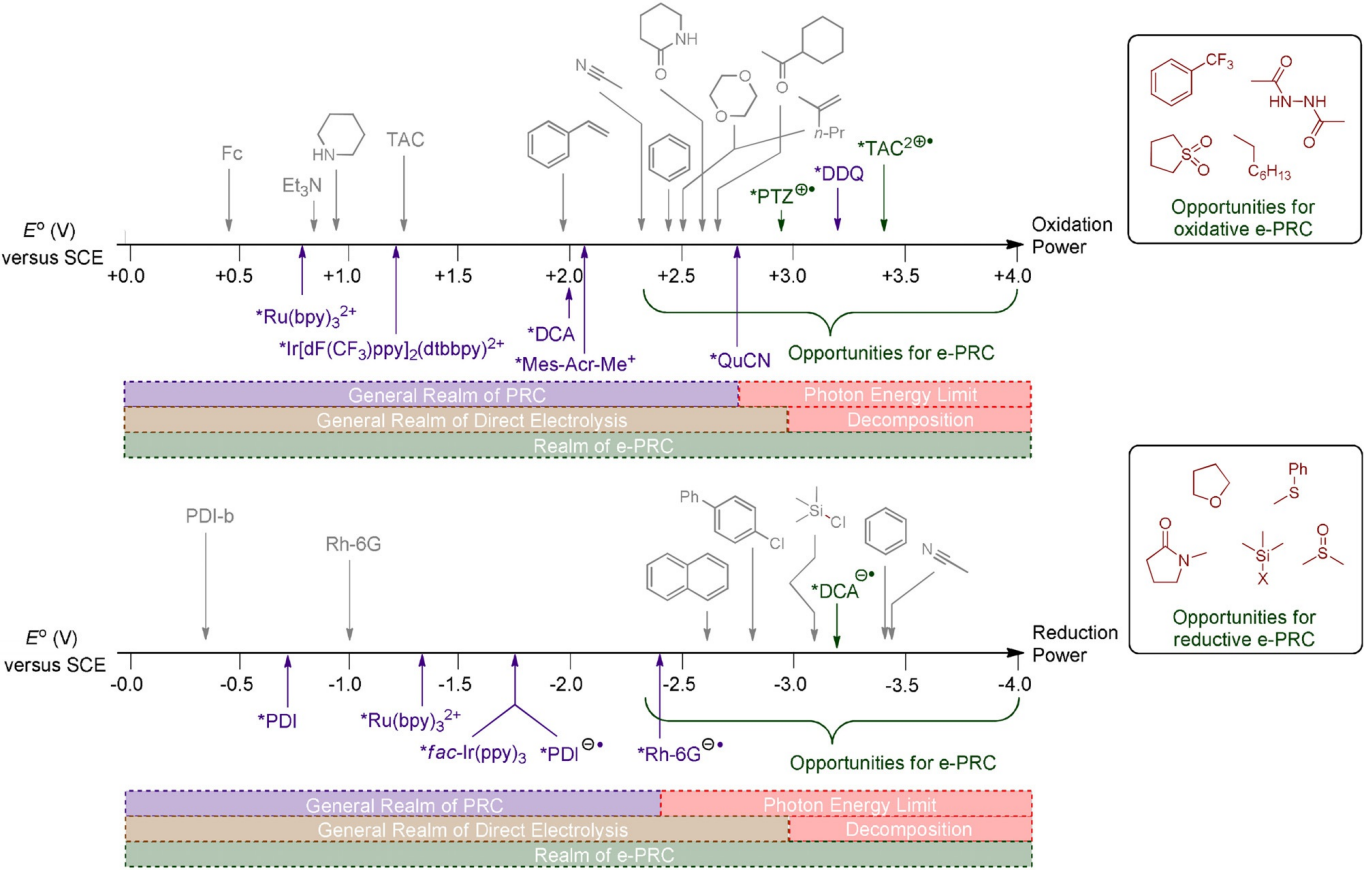
Redox potential scale showing current limitations of PRC and direct electrolysis technologies and opportunities for e‐PRC.^86^

The elucidation of such precomplexation mechanisms presents a challenge and demands the use of advanced spectroscopic, spectroelectrochemical, and theoretical (computational) tools.^84^, ^85^ The ability to generate super‐oxidants and super‐reductants in situ and within close proximity to the substrate of interest (by an e‐PRC–substrate precomplexation) may allow redox processes to take place at potentials beyond those available from PRC and beyond those normally tolerable by the organic solvent in which the reaction takes place (Figure 20). Thereby, e‐PRC may create a “realm” for extremely challenging SET processes such as direct oxidations of carbonyl compounds, sulfones, fluorinated aromatics, and hydrocarbons. Direct reductions of amides, ethers, Si−X bonds (X=Cl, F, OSiR_3_, OR), sulfoxides, and sulfides may be possible. The potentials that would be required in such scenarios by SoE would no doubt lead to decomposition/poor chemoselectivity. Finally, a notable challenge is the inability to measure redox potentials of substrates that lie beyond the redox window of the solvent.^86^ Here, computational methods^87^ to estimate redox potentials may prove useful.

### 3.4 Nomenclature

One key challenge for the scientific community is the adoption of appropriate and consistent nomenclature in this rapidly developing field of synthetic photoelectrochemistry. This is important not only to avoid misunderstanding between the different strategies and concepts herein, but also to avoid confusion with other distinct research fields and phenomena such as photoelectrolysis and the photoelectric effect. In fact, reports discussed herein (that do not involve photoelectrodes) are divided in their use of the terms “photoelectrochemistry”,^47^ “photoelectrocatalysis”,^50^ and “electrophotocatalysis”,^38^, ^39^, ^49^ while others carefully avoided using such terms.^52^

The IUPAC definition^88^ of “photoelectrochemistry” is a *“term applied to a hybrid field of chemistry employing techniques which combine photochemical and electrochemical methods for the study of the oxidation‐reduction chemistry of the ground or excited states of molecules or ions. In general, it is the chemistry resulting from the interaction of light with electrochemical systems*.” Therefore, we recommend ”synthetic photoelectrochemistry“ as a blanket term that can encompass all of the examples presented herein.

In electrochemistry, “electrocatalysis” refers to catalysis of electrochemical reactions by surface states of electrodes (e.g., Pt is an electrocatalyst for proton reduction to H_2_),^89^ where the role of catalysis is to lower the activation energy barrier for heterogeneous electron transfer. Accordingly, “photoelectrocatalysis” and “electrophotocatalysis” have historically referred to the catalysis of electrochemical reactions on the surface states of photoelectrodes. To specify the interfacial nature of this chemistry, as in Section 2.4, we recommend the terms “interfacial photoelectrochemistry”60a or “interfacial photoelectrocatalysis”.^57^

“Redox catalysts” and “mediators” are well‐established terms in SOE for the shuttling of electrons between electrode surfaces and substrates,^21^, ^89^ while “photoredox catalysis” is a ubiquitous term in its field.^2^, ^4^ All examples in Section 2.1 can be considered as “photoredox catalysis”, either at an elevated redox energy level or with electrochemical turnover. The catalysis of most interest to the synthetic chemist (that which is depicted in every example)^38^, ^39^, ^47^, ^49^, ^50^ is indeed the photocatalytic cycle of the redox catalyst, *not* catalysis of the interfacial reaction involved in its generation/regeneration. In light of all of the above, we recommend the terms “electrochemically mediated photoredox catalysis (e‐PRC)” and “electromediated photoredox catalysts” (e‐PRCs).

## 4 Summary and Outlook

Synthetic photoelectrochemistry is a swiftly emerging research field following renaissances in its respective parent technologies, photoredox catalysis (PRC) and synthetic organic electrochemistry (SOE) that have taken place over the last decade.^90^, ^91^ To simplify the technology for users, this Review sets precedent for grouping historic and recent reports into three categories of photoelectrochemistry: electrochemically mediated photoredox catalysis (e‐PRC), decoupled photoelectrochemistry (dPEC), and interfacial photoelectrochemistry (iPEC). The fundamental advantages that derive from the fusion of PRC and SOE are expected to 1) broaden the accessible redox window of SET chemistry,^35^, ^38^, ^39^ 2) enable milder conditions that allow greater functional group tolerance and chemoselectivity,^49^, ^52^, ^57^ and 3) increase energy savings and atom economy.^60^, ^64^ Practical challenges in the execution of synthetic photoelectrochemistry could be addressed by an equipment and expertise interface with research fields of photoelectrochemical cells for water splitting and photovoltaic cells, while flow chemistry is expected to offer significant benefits to the transmission of light/electrons,^76^, ^77^ kinetics, and scalability of photoelectrochemical reactions.^76^, ^77^ We are particularly excited by the concept of an e‐PRC–substrate precomplexation.11d, 83 Further understanding of this concept is of critical importance, with potential to leverage it to improve the kinetics of SET processes as well as to control redox chemoselectivity.

## Conflict of interest

The authors declare no conflict of interest.

## Biographical Information

*Joshua P. Barham was born in Watford (UK). He received his industry‐based PhD in 2017 under the supervision of Prof. John A. Murphy at the University of Strathclyde and Dr. Matthew P. John at GSK (UK). His postdoctoral studies with Prof. Yasuo Norikane and Prof. Yoshitaka Hamashima at AIST and the University of Shizuoka (Japan) specialized in photoredox catalysis and microwave flow chemistry. In 2019, he was awarded a Sofja Kovalevskaja Award from the Alexander von Humboldt foundation to lead an independent research group at the University of Regensburg, investigating photo‐, electro‐, photoelectro‐, and flow chemistry in organic synthesis*.

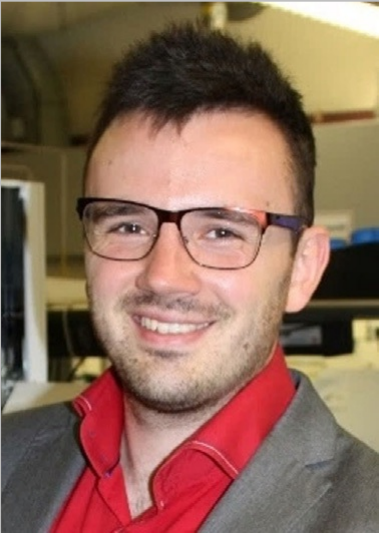

## Biographical Information

*Burkhard König was born in Wiesbaden (Germany). He obtained his PhD in 1991 from the University of Hamburg and pursued postdoctoral studies with Prof. M. A. Bennett, Research School of Chemistry, Australian National University, Canberra, and Prof. B. M. Trost, Stanford University. He became full professor of organic chemistry at the University of Regensburg in 1999. His current research interests revolve around the application of visible‐light chemical photocatalysis towards organic synthesis*.